# Correlates for psycho-active substance use among boarding secondary school adolescents in Enugu, South East, Nigeria

**DOI:** 10.1186/s12887-016-0615-9

**Published:** 2016-06-09

**Authors:** Pius C. Manyike, Josephat M. Chinawa, Awoere T. Chinawa, Herbert A. Obu, Ada R.C. Nwokocha, Odutola I. Odetunde

**Affiliations:** College of Medicine, Ebonyi State University/Department of Pediatrics, Federal Teaching Hospital, Abakiliki, Nigeria; Department of Pediatrics, College of Medicine, University of Nigeria, Enugu Campus/University of Nigeria Teaching Hospital (UNTH), Ituku- Ozalla, Enugu State 400001 Nigeria; Department Of Community Medicine, College of Medicine, Enugu State University Teaching Hospital, Enugu, 400001 Enugu Nigeria

**Keywords:** Adolescents, Psychoactive substance use, Nigeria

## Abstract

**Background:**

Psycho-active substance use among adolescents is a national and global problem and its attendant effects on adolescents cannot be overemphasized.

The objectives of this study are to determine the prevalence and pattern of psychoactive substance use among adolescents; the substances involved and the extent of the problem in this locale.

**Methods:**

This is a cross-sectional study that assesses the pattern of psychoactive substance use among secondary school adolescents in Enugu, south East, Nigeria. The study was carried out among adolescents attending six secondary boarding schools in Enugu metropolis of Enugu State of Nigeria.

The WHO Student Drug Use Questionnaire was adapted for this study.

Data were analyzed using the Statistical Package for Social Sciences program (SPSS), version 17. Chi-square and multivariate regression were used as a test of significance for qualitative variables. A *p*-value less than 0.05 were accepted as significant for each statistical test.

**Results:**

Out of 900, a total of 896 respondents, comprising 400 and 82 boys (482) (53.8 %) and 400 and 14 girls (414) (46.2 %) completed the questionnaires. This gave a response rate of 99.6 %.

The study revealed that the prevalence of current use for psychoactive substances ranges from 0.4 to 34.9 % while that for life use ranges from 0.8 to 63.5 %. The least being cannabis and the most being kola nuts.

Kola nut is the most widely used psychoactive substance both for current use, past year use and the respondents’ life time use. It shows a lifetime prevalence of 63.5 % and a current use prevalence of 34.9 %.

More than half of the users of each of the psychoactive substances take it occasionally, using them on 1–5 days in a month. On the other hand, almost one-quarter of the users of each of the substances take it on 20 or more days in a month.

**Conclusion:**

The study revealed that the prevalence of current use for psychoactive substances ranges from 0.4 to 34.9 % while that for life use ranges from 0.8 to 63.5 %. The least being cannabis and the most being kola nuts.

**Electronic supplementary material:**

The online version of this article (doi:10.1186/s12887-016-0615-9) contains supplementary material, which is available to authorized users.

## Background

Psychoactive substance use constitutes a major public health and social problem worldwide, with alcohol, tobacco and marijuana being the most commonly used. These substances are used for medicinal, social and religious purposes [1, 2].

In Nigeria, the National Drug Law Enforcement Agency, NDLEA, was established to curb the menace of substance use and misuse [2]. However, there are still reported increases in the trend of substance use, particularly among adolescent [3, 4].

Psychoactive substance use among adolescents is a national and global problem. For instance, findings from the national representative samples of US youths revealed that the lifetime prevalence of alcohol use disorders is approximately 8 % and that for other substance use is 2–3 % [5, 6]. The striking increase in prevalence rates from the age of 13 to 18 years highlights adolescence as the key period of development of substance use disorders (SUDs) [5, 6].

Adolescents who use psychoactive substances run the risk of increased criminality before age twenty with frequent mental health referrals [7–9]. The use of psychoactive substances adversely affects the physical, cognitive, emotional, behavioral, social and spiritual development of the adolescents [10, 11]. It may, for instance, be associated with public violence, financial problems, sexual difficulties and family disruption [12, 13].

Psychiatric manifestations may also arise from these substance use. This includes panic spells, flashbacks, psychosis, homicides, suicidal thoughts and dependence [14, 15].

Instances of substance use have also been reported in Nigeria. For instance, Oluwale et al. [16] in their work, for example, noted that 61.8 and 32.1 % of respondents have used one or more psychoactive substances in their lifetime and in the past 1 year. In Enugu, Igwe et al. [17] noted that psychoactive substances most commonly used were alcohol (31.6 %), cola nitida (kola nut) (20.7 %) and coffee (15.7 %).

Similar studies done in Nigeria by Oshodi et al. [18], Ene et al. [19] were about a decade ago while Igwe and Ojinnaka et al. [20, 21] did theirs about 5 years ago, however much has not been done on this topic in the past 5 years. Moreover, a careful search also showed that the work done by the above authors [18–20] was not principally among adolescents who live in boarding schools.

This study, therefore, is aimed at determining the correlates and pattern of psychoactive substance use among adolescents in boarding schools. It is hoped that the findings of this study will add to the already existing body of knowledge, and will reveal the present trend in substance use. This will also serve as a template for future studies.

### Definition of terms

Kola nut with caffeine [22] as the active ingredient is from the plant species- Cola accuminata and Cola nitida [22]. Coffee is a mild stimulant and like kola nut contains caffeine [22]. It is derived from coffea canephora which occurs commonly as shrub in the forest of southern Nigeria.

## Methods

### Study design

This is a cross-sectional study that assessed the pattern of psychoactive substance use among adolescents attending boarding schools in Enugu, south East, Nigeria.

### Study area

The study was carried out among adolescents aged between 15 years to 19 years attending six boarding secondary schools in Enugu metropolis.

Enugu, the capital of Enugu state of Nigeria was chosen for this study because it has many secondary schools with large adolescent population. It is made up of three local government areas, namely, Enugu East, Enugu North and Enugu South. There are 27 secondary schools in Enugu metropolis with a total population of 94,401 students^’^. These schools are evenly distributed. The students’ population of these schools cuts across the various socio-economic strata in Nigeria. These schools were stratified into boys, girls, and mixed schools in order to get a representative sample. There are nine boys, ten girls and eight mixed schools.

Nigerian secondary education is organized into two major categories: Junior and Senior Secondary Schools each lasting for 3 years. The boarding schools are either mixed, “boys” only, or “girls” only.

### Questionnaire

The WHO (World Health Organization) Student Drug Use Questionnaire [23] was adapted for this study. It was originally developed by the World Health Organization in collaboration with the United Nations Fund for substance use in different socio-cultural settings. The instrument was shown to have satisfactory validity [24].

The prototype questionnaire (WHO questionnaire, Geneva for substance use) is made up of 22 items altogether, comprising those in demographic characteristic (6 items), frequency and age at first use of ten types of psychoactive substances including alcohol and tobacco (14 items), and the honesty with which the questions were answered (2 items). See Additional file 1.

The items on parents’ level of education and occupation as well as that on respondents’ monthly pocket money were incorporated into the questionnaire as measures of socio-economic status. Those on the age of the respondents at the time of divorce or death of their parents (if applicable) were aimed at estimating the proportion of students who suffered some degree of parental deprivation.

The questionnaire which was self administered was completed by the students after explanation of the purpose of the study. Confidentiality was assured by informing the respondents not to write their names on the questionnaire.

Adolescents who gave consent were included in this study while those who did not were excluded.

### Pilot study

A pre-test was done using Command Secondary School, Abakpa Nike, Enugu, because of the ethnic and socio-cultural diversity of the students’ population. The aim was to identify and remove ambiguities in the survey instrument.

One hundred and fifty students participated in the pre-test. This comprised 50 students from each class of Senior Secondary 1 to Senior Secondary 3. The exercise was done after full explanation of the purpose of the exercise and the fact that confidentiality was assured. Questions and difficulties that arose during the exercise were fully explained. The average questionnaire completion time was 50 min with a range of 40–60 min. The researcher was present while the questionnaire was being completed. The teachers were, however, excluded from the hall. The entire questionnaire was completed and returned. On the whole, the questionnaire was adjudged valid and reliable.

### Study procedure

Six schools were selected out of the twenty seven schools in Enugu metropolis through a stratified random sampling method. This comprised two boys’ only schools, two girls’ only schools and two mixed schools.

By stratified random selection method, a total of 150 students at each boys’ and girls’ school were selected by class stream.

### Data analysis

All data were coded, entered, and analyzed using the Statistical Package for Social Sciences program (SPSS), version 17. Results were presented in cross tabulations and histogram. Chi-square was used to test significant association for qualitative variables while multivariate logistic regression was used to determine correlates. A *p*-value less than 0.05 was accepted as significant for each statistical test.

## Results

### Types of substances used

Nine hundred questionnaires were distributed. Eight hundred and ninety six of the questionnaires were analyzed, while four questionnaires were not properly filled. This gave a response rate of 99.6 %. Their age range was 15 years to 19 years with a mean of 15.9 ± 1.04 years.

The male respondents in this study were 482 (53.8 %), while the female were 414 (46.2 %). Most of the respondents (99.1 %) are Christians and 78.7 % of them actively participate in religious activities as shown in Table 1. Ninety-one point one percent (91.1 %) of the respondents’ fathers and (96.4 %) of their mothers are alive. Only 14.4 % of the respondents have parental deprivation. Parental psychoactive substance use shows that 62 (6.9 %) of the fathers smoke and 3 (0.3 %) of the mothers smoke. Also 306 (34.2 %) of the fathers take alcohol and 130 (14.5 %) of the mothers take alcohol.

**Table 1:** Respondents’ demographic and parental background

Characteristics	Frequency	Percentage
Sex		
Male	482	53.8
Female	414	46.2
Religion		
Christianity	888	99.1
Islam	6	0.7
Traditional African Religion	2	0.2
Parental Background		
Father dead	80	8.9
Mother dead	32	3.6
One or both parents dead	106	11.8
Parents alive but divorced	23	2.6
Parents deprivation	129	14.4
Father smokes	62	6.9
Mother smokes	3	0.3
Father takes alcohol	306	34.2
Mother takes alcohol	130	14.5

In Table 2, five hundred and sixty nine or 63.5 % of the population have ever taken kolanut. This implies that Kola nut is the most widely used psychoactive substance both for current use, past year use and the respondents’ life time use. It shows a lifetime prevalence of 63.5 % and a current use prevalence of 34.9 %. The same table revealed that the prevalence of current use for the substances ranges from 0.4 to 34.9 % while that for life use ranges from 0.8 to 63.5 %. The least being cannabis and the most being kolanuts.

**Table 2:** Prevalence of past year user, current user and lifetime user of substances

Substances	Past year use *N* = 896	Current use *N* =896	Lifetime use *N* = 896
Kolanut	457 (51.0 %)	313 (34.9 %)	569 (63.5 %)
Alcohol	435(48.5 %)	183 (20.4 %)	531 (59.3 %)
Coffee	159 (17.7 %)	121 (13.5 %)	176(19.6 %)
Tobacco	55 (6.1 %)	26 (2.9 %)	92 (10.3 %)
Tranquillizers	63 (7.0 %)	42(4.7 %)	90 (10.0 %)
Cannabis	7 (0.8 %)	4 (0.4 %)	7 (0.8 %)

**Fig. 1:**
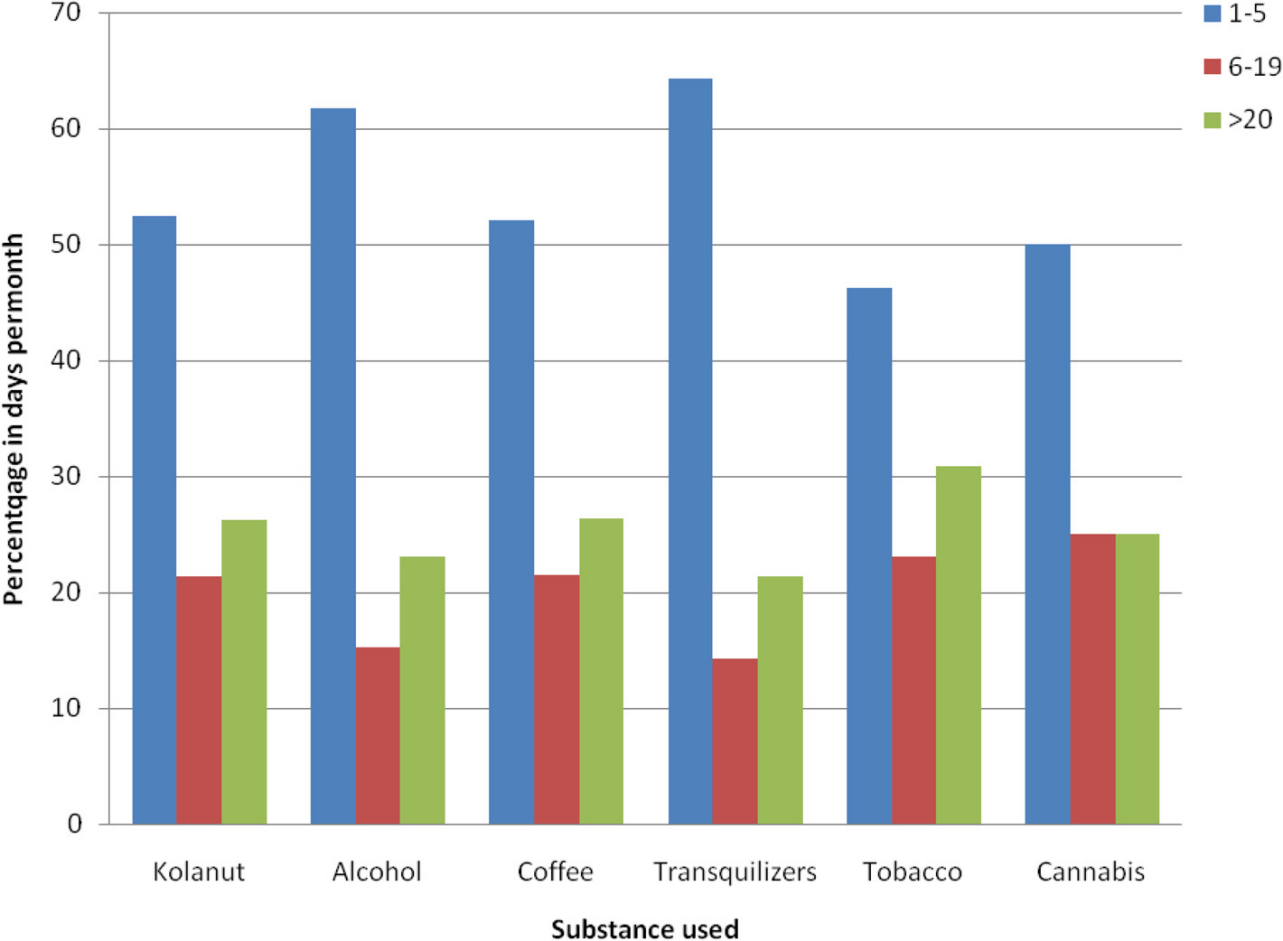
Frequency of drug use in days per month based on current use

Figure 1 show that among the current users of the psychoactive substances, more than half of the users of each of the substances take it occasionally, using them on 1–5 days in a month. On the other hand, almost one-quarter of the users of each of the substances take it on 20 or more days in a month. However, the frequency in days per month based on the current use does not depend on the substances used (*P* > 0.05).

Table 3 shows more male involvement in this substance life time use (*P* < 0.05). Cannabis was not used by any of the female respondents. However, more males than females have used all the substances.

**Table 3:** Prevalence of life time use of the various substances base on gender (sex)

Substances	Male (*n* = 482)	Female (*n* = 414)
Kolanut	474 (98.3 %)	95 (22.9 %)
Alcohol	298 (61.8 %)	233 (56.3 %)
Coffee	133 (27.6 %)	26 (6.3 %)
Tranquillizers	49(10.2 %)	41 (9.9 %)
Tobacco	85 (17.6 %)	7 (1.7 %)
Cannabis	7 (1.5 %)	0 (0.0 %)

Table 4 shows that psychoactive substance use based on life time use is dependent on the age at first use (*P* < 0.05). Majority of the respondents first used Kolanut and coffee at the age less than 10 years, alcohol, tranquilizers and tobacco at the age of 11–14 years, while cannabis at the age of 15–19 years.

**Table 4:** Respondents age ranges at first use of various substances based on life time use

Substances	Age at First Use (years)		
	≤10	11–14	15–19
Kolanut (*n* = 569)	235 (41.3 %)	222 (39.0 %)	112 (19.7 %)
Alcohol (*n* = 531)	192(36.2 %)	249 (46.9 %)	90 (16.9 %)
Coffee (*n* = 159)	70 (44.0 %)	66 (41.5 %)	23 (14.5 %)
Tranquillizers (*n* = 90)	24 (26.7 %)	51(56.7 %)	15 (16.7 %)
Tobacco (*n* = 92)	13 (14.1 %)	44 (47.8)	35 (38.0 %)
Cannabis (*n* = 7)	0 (0.0 %)	2 (28.6 %)	5 (71.4 %)

*χ* ^2^_=_62.125 df = 10 *p*-value =0.000

Table 5 shows that psychoactive substance use based on the life time use is dependent on the level of education at first use *p* = 0.05. Majority of the respondents first used Kolanut and coffee in primary school, while alcohol, tranquilizers, tobacco and cannabis were used in secondary school.

**Table 5:** Respondents level of education at first use of various substances based on life time use

Substances	Level of Education at first use	
	Primary School	Secondary School
Kolanut (*n* = 569)	334 (58.7 %)	235 (41.3 %)
Alcohol (*n* = 531)	221(41.6 %)	310(58.4 %)
Coffee (*n* = 159)	88(55.3 %)	71 (44.7 %)
Tranquillizers (*n* = 90)	29(32.2 %)	61 (67.8 %)
Tobacco (*n* = 92)	12(13.0 %)	80 (87.0 %)
Cannabis (*n* = 7)	0 (0.0 %)	7 (100.0 %)

*χ* ^2^_=_98.479 df =5 *p*-value =0.000

Table 6 shows no significant association between sex, age, class frequency of usage, father’s education and the respondents’ use of alcohol (*P* > 0.05); except mothers education and the use of alcohol (*P* = 0.002) Table 7

**Table 6:** Association between respondents’ lifetime, previous year and current use of alcohol with social class and demographic variables

Variables	Use of alcohol		
	Lifetime use	Previous year use	Current use
Sex			
Male	298 (33.3 %)	248 (27.7 %)	108 (12.1 %)
Female	233 (26.0 %)	187 (20.9 %)	75 (8.4 %)
Age			
15	184 (20.5 %)	145 (16.2 %)	64 (7.1 %)
16	182 (20.3 %)	153 (17.1 %)	67 (7.5 %)
17	116 (12.9 %)	96 (10.7 %)	37 (4.1 %)
18	37 (4.1 %)	32 (3.6 %)	10 (1.1 %)
19	12 (1.3 %)	9 (1.0 %)	5 (0.6 %)
Class			
SS 1	135 (15.1 %)	113 (12.6 %)	44 (4.9 %)
SS 2	212 (23.7 %)	172 (19.2 %)	82 (9.2 %)
SS 3	184 (20.5 %)	150 (16.7 %)	57 (6.4 %)
Fathers Education			
Below Sec. Sch.	229 (25.6 %)	187 (20.9 %)	66 (7.4 %)
Above Sec. Sch.	302 (33.7 %)	248 (27.7 %)	117 (13.1 %)
Mothers Education			
Below Sec. Sch.	242 (27.0 %)	195 (21.8 %)	73 (8.1 %)
Above Sec. Sch.	289 (33.3 %)	240 (26.8 %)	110 (12.2 %)

**Table 7:** Logistic regression of the relationship between respondents’ lifetime, previous year and current use of alcohol with social class and demographic variables

Variables	B	S.E.	Wald	df	*P*-value	Exp (B)	95 % C.I. for Exp (B)	
							Lower	Upper
Lifetime Use								
Sex	−.212	.146	2.120	1	.145	.809	.617	1.005
Age	−.521	.080	42.474	1	.000	.594	0.511	.679
Class	.769	.113	45.963	1	.000	2.158	2.009	2.310
Frequency	.288	.148	3.801	1	.051	1.334	1.277	1.399
f.edu	.264	.151	3.066	1	.080	1.302	1.283	1.325
m.edu	−.036	.151	.058	1	.809	.964	.938	.992
Constant	7.191	1.213	35.152	1	.000	1327.730		
Previous year Use								
Sex	−.269	.140	3.721	1	.054	.764	.698	.834
Age	−.242	.074	10.609	1	.001	.785	.736	.841
Class	.143	.102	1.960	1	.162	1.154	1.112	1.199
Frequency	.321	.139	5.333	1	.021	1.379	1.307	1.153
f.edu	.212	.145	2.154	1	.142	1.236	1.198	1.279
m.edu	−.061	.145	.175	1	.676	.941	.898	.991
Constant	3.652	1.143	10.202	1	.001	38.537		
Current Use								
Sex	−.289	.175	2.730	1	.098	.749	.713	.787
Age	−.414	.098	17.687	1	.000	.661	.635	.690
Class	.303	.124	5.947	1	.015	1.354	1.288	1.421
Frequency	.117	.167	.489	1	.484	1.124	1.093	1.159
f.edu	.273	.182	2.246	1	.134	1.314	1.276	1.352
m.edu	−.119	.182	.426	1	.514	.888	.842	.933
Constant	4.836	1.501	10.382	1	.001	125.934		

.

Table 7 shows stepwise logistic regression (95 % CI logistic regression of the relationship between respondents’ lifetime, previous year and current use of alcohol with social class and demographic variables). Age and class have significant relationship to the respondents’ lifetime use of alcohol (*P* = 0.000 and *P* = 0.000 respectively), while age and frequency have significant relationship to the respondents’ past year use of alcohol (*P* = 0.001 and *P* = 0.021 respectively), and age and social class have significant relationship to the respondents’ current use of alcohol (*P* = 0.000 and *P* = 0.015 respectively).

## Discussion

The present study showed kola nut, alcohol, tranquillizers, tobacco and cannabis as the substances used by the adolescents studied. These findings, agreed with the findings of previous studies in Ilorin Nigeria [25, 26]. None of the respondents in the present study admitted having used amphetamines, cocaine, heroin, hallucinogens, opiates, barbiturates or volatile agents. This is in keeping with earlier reports where it was found that the use of these agents are not yet common among adolescents in Nigeria, though, some few cases have been reported [25, 26].

The present study showed that the prevalence of current use for the substances ranges from 0.4 to 34.9 % while that for lifetime use ranges from 0.8 to 63.5 %. The least being cannabis and the most being kola nuts. The value is lower than those of previous studies. For instance, results from 2010, in the United States, showed that 48.2 % of adolescents reported having used psychoactive substances at some point in their lives [27, 28]. Some methodological differences have been identified as affecting these prevalence rates. This includes populations covered, sampling methods, and mode of data collection, survey setting, questionnaire, and estimation methods [28, 29].

Kola nut emerged the most commonly used psychoactive substance in this study both in the respondents’ life time and the month preceding the study. This is because adolescents use kola nut to achieve long hours of wakefulness during examinations.

Alcohol in the present survey is the second most commonly used psychoactive substance. Earlier reports by Akpala and colleagues 1991 in Nigeria have also shown alcohol to be the second most used psychoactive substance [30]. It is pertinent to note that alcohol is widely available in Nigeria and easily accessible to various age groups. Alcohol did not show a similar trend as the other substances that showed a decline as the respondents moved from primary school to secondary school.

The fact that there is a positive correlation between respondents’ alcohol use and use by the parents as shown in this study is also supported by Cleveland et al. [31]. We noted that age and socio economic class of subjects have significant relationship to the respondents’ lifetime and current use of alcohol. It was noted that some mechanism could explain this variations of socioeconomic class, age and substance abuse. For instance, increased risk of alcohol use and related psychosocial problems with greater household income may reflect greater availability in such households or greater ‘purchasing power’ among children from such households. There may also be cultural norms to explain different alcohol behaviors across the socio-economic spectrum [32, 33].

When multivariate logistic regression was used, we noted that age and socio economic class of subjects have significant relationship to the respondents’ lifetime and current use of alcohol, thus, supporting the result of earlier study [31].

Religious participation also shows a positive correlation with respondents’ intake of alcohol. A study conducted in Ireland, suggested that university students who attended religious services infrequently and university students who did not believe in God reported using more alcohol [34]. Similarly, another study in Australia found that students who believed that religion was unimportant in their lives reported using more alcohol, tobacco, marijuana and hallucinogens.

Dalgalarrondo et al. [35]. Furthermore, a study conducted in a university in the United States, showed that the use of ecstasy was higher among students who stated that religion had little influence on their lives [34].

In Brazil, few studies have focused on the relationship between religious involvement and substance use. The majority of the available studies have found that religiosity is protective against substance use in samples of Brazilian adolescents and a few studies have focused on this relationship among university students [34, 36].

Tobacco, the third psychoactive substance mostly used by the students showed a similar prevalence with that recorded in Kano (In northern Nigeria) by the NDLEA [25]. Tobacco in the form of snuff (powdered form) is enjoyed by most parents. Only few of the respondents reported use of snuff because tobacco use in the form of snuff is not fashionable among the adolescents. The adolescents who admitted using them in this study were only those whose parents use it and who only experimented with it in their homes.

In the Southern parts of the country, people frown at smoking by adolescents hence the low prevalence for both lifetime and current uses obtained in this study. It is noteworthy that result of the present study on cigarette smoking is lower than those obtained in Mexico, England and South Africa [37]. In Mexico and England, cigarette smoking is socially accepted while the high prevalence obtained in the black township of South Africa could be explained by the stress in the then apartheid South Africa which made the black boys to smoke. We also noted in this study that smaller percentage of the respondents smoked before the age of 10 years. This reflects mainly family influence, both parental and sibling.

Tranquillizers are the fifth most commonly used psychoactive substance. The current use prevalence is almost in agreement with NDLEA’s 3.5 % [25]. It is also important to note that students use tranquillizers such as diazepam to induce sleep after using stimulants to keep awake. Furthermore, we did not obtain any statistically significant difference between male and female involvement in the use of tranquillizers as against the work of Emerita et al. who showed female preponderance in the use of tranquillizers [37]. The reason for this could be due to the small sample size used by Emerita. Racial and socio-cultural differences could also explain these gender differences. Today, the commonest psychoactive substance that is found in most home medicine stores is tranquillizers. This is not unconnected with the level of stress in the society. The fact that some of the respondents used tranquillizers before 10 years of age and in primary school shows the influence of the family in introducing their children to the use of this substance.

Cannabis is the least substance used. The current prevalence almost agreed with Adelekan’ s 0.5 % but differs from the high values obtained in Sokoto, Kano and Lagos (North and south west Nigeria) [25]. The low value obtained in this study may be due to under-reporting because not only that the use of cannabis is associated with psychosis and criminal activities, but that the society frowns at its use. The law in Nigeria also prohibits both the use and sales of cannabis. Though the abuse of cannabis is low, it is gradually increasing over the years [38]. Moreover, we noted that only male respondents admitted using cannabis and that all of them started the habit in secondary school. The fact that none of the female respondents admitted using cannabis in this study contradicts the reports of Faeh et al. who found an increasing female involvement in the use of cannabis [38]. It is also noted in this study that while the use of the other substances except alcohol declined as the ages of the respondents increased that of cannabis increased as their ages increased. This implies that most of the respondents give up some of their psychoactive substance use habits as they mature whereas those of them who use cannabis had their number increased instead of declining probably because they started the habit late and are yet to give it up.

Boarding schools tend to enroll a high concentration of high risk or problem prone youths who create risk-taking peer networks [39]. It is noted that many of the adolescents who enroll in these schools have experienced considerable family chaos, violence, stress, and severe disruption in the formative years of their lives. Barrera et el 2001 [39] in India pointed out that boarding students who are exposed to such substances are usually not monitored by their parents at home and for some who do, are away from home for long periods.

In all, our study showed that none of the respondents is a problem drinker, majority of them drink about half a beer bottle of alcohol daily. In the same vein, none of the respondents is a heavy smoker, majority of them smoke one stick of cigarette a day. This study however, showed that a fraction of the respondents have tried to stop or reduce the use of these substances but were unable to do so. These are the respondents who have developed dependence on these drugs. About half of the respondents were initiated into psychoactive substance use by their friends, while father and mother accounted for a third and a fifth respectively. The value does not differ significantly from the 40 % obtained by NDLEA [24]. The fact that none of the respondents answered the question on the fictitious psychoactive substance showed that over-reporting is not a problem in the present study.

### Strength and weaknesses of the study

The sample has its strength in the sample size and number of schools selected. For the fact that this is the first time over half a decade (as much as we know) this study is carried out among adolescents in this vicinity, is worthwhile. The weakness of this study lies in the fact that a cross-sectional survey was done. A longitudinal study would have permitted the evaluation of the changing pattern of psychoactive substance use over time.

## Conclusion

The study revealed that the prevalence of current use for the psychoactive substances ranges from 0.4 to 34.9 % with kola nut emerging as the most commonly used psychoactive substance. There is, however, positive correlation between year of study, participation in religious activities, parental educational background and respondents’ use of substance.

## Abbreviations

NDLEA, National Drug Law Enforcement Agency; SPSS, statistical package for social sciences program; SUDs, substance use disorders; WHO, World Health Organization

## Additional file

Additional file 1: WHO questionnaire, Geneva for substance use (DOCX 714 kb)
