# Supplementary figures and images for: Correlates for psycho-active substance use among boarding secondary school adolescents in Enugu, South East, Nigeria
Source: BMC Pediatr. 2016 Jun 9;16:78. doi: 10.1186/s12887-016-0615-9 (PMC4899923; doi:10.1186/s12887-016-0615-9)

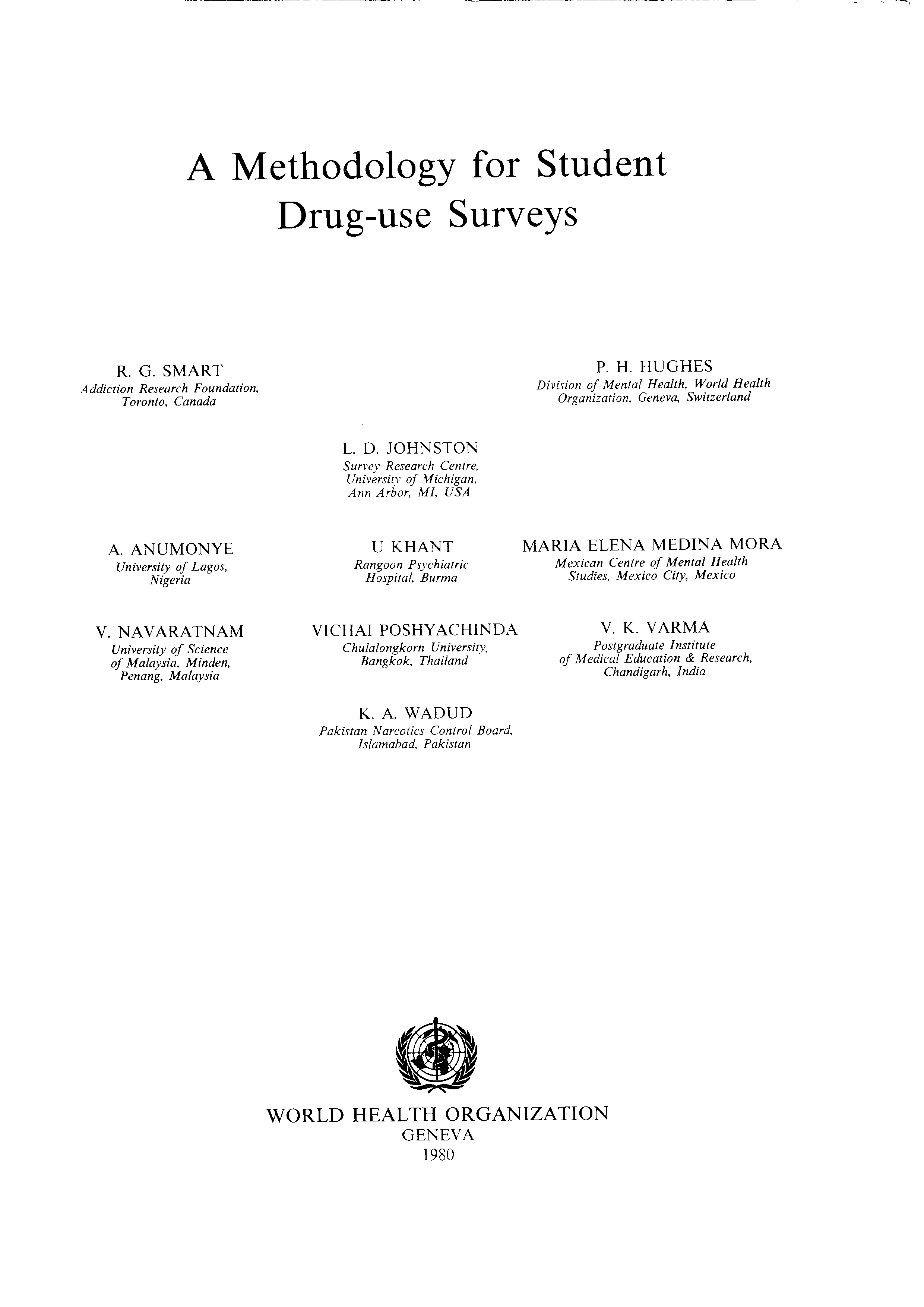

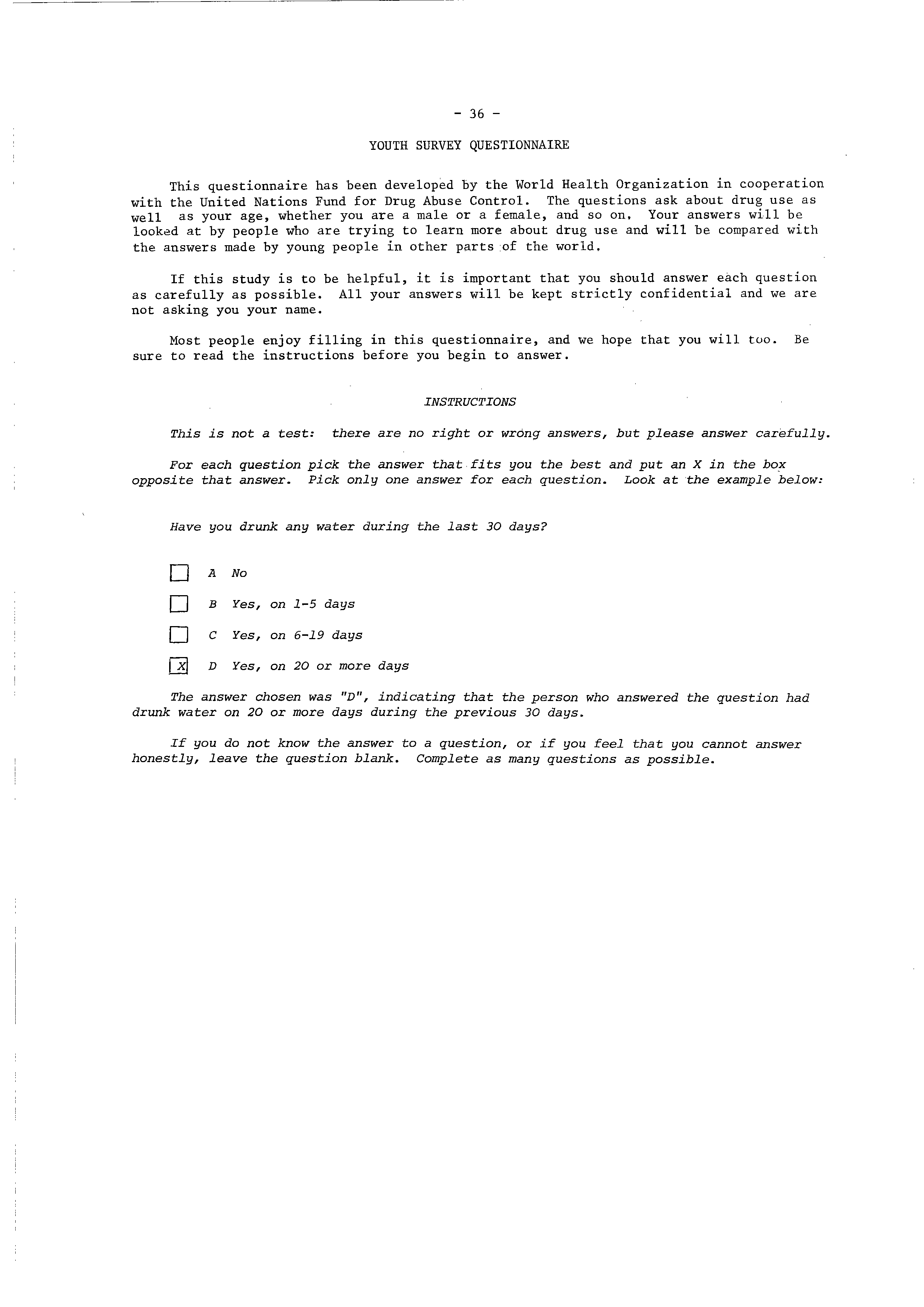

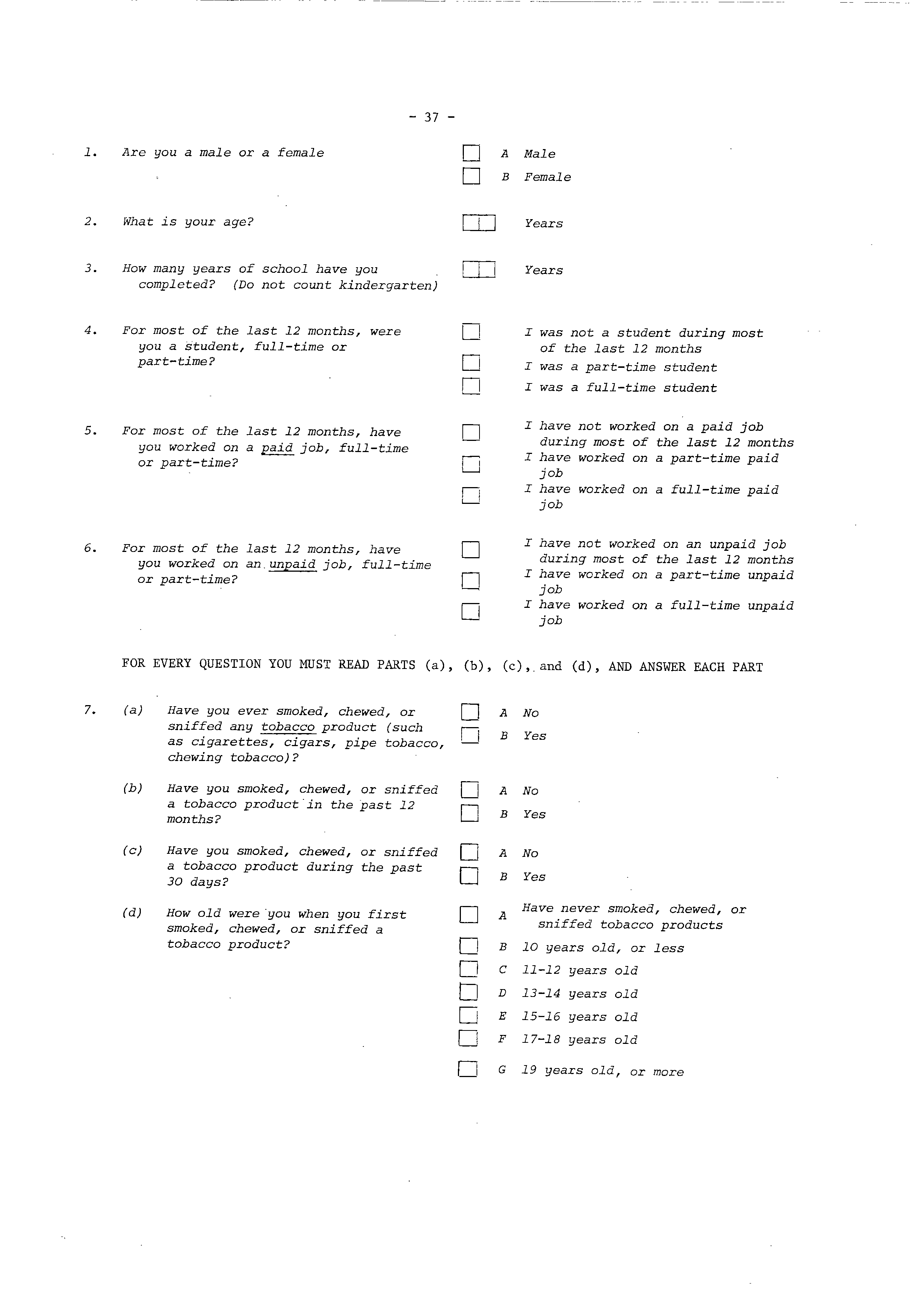

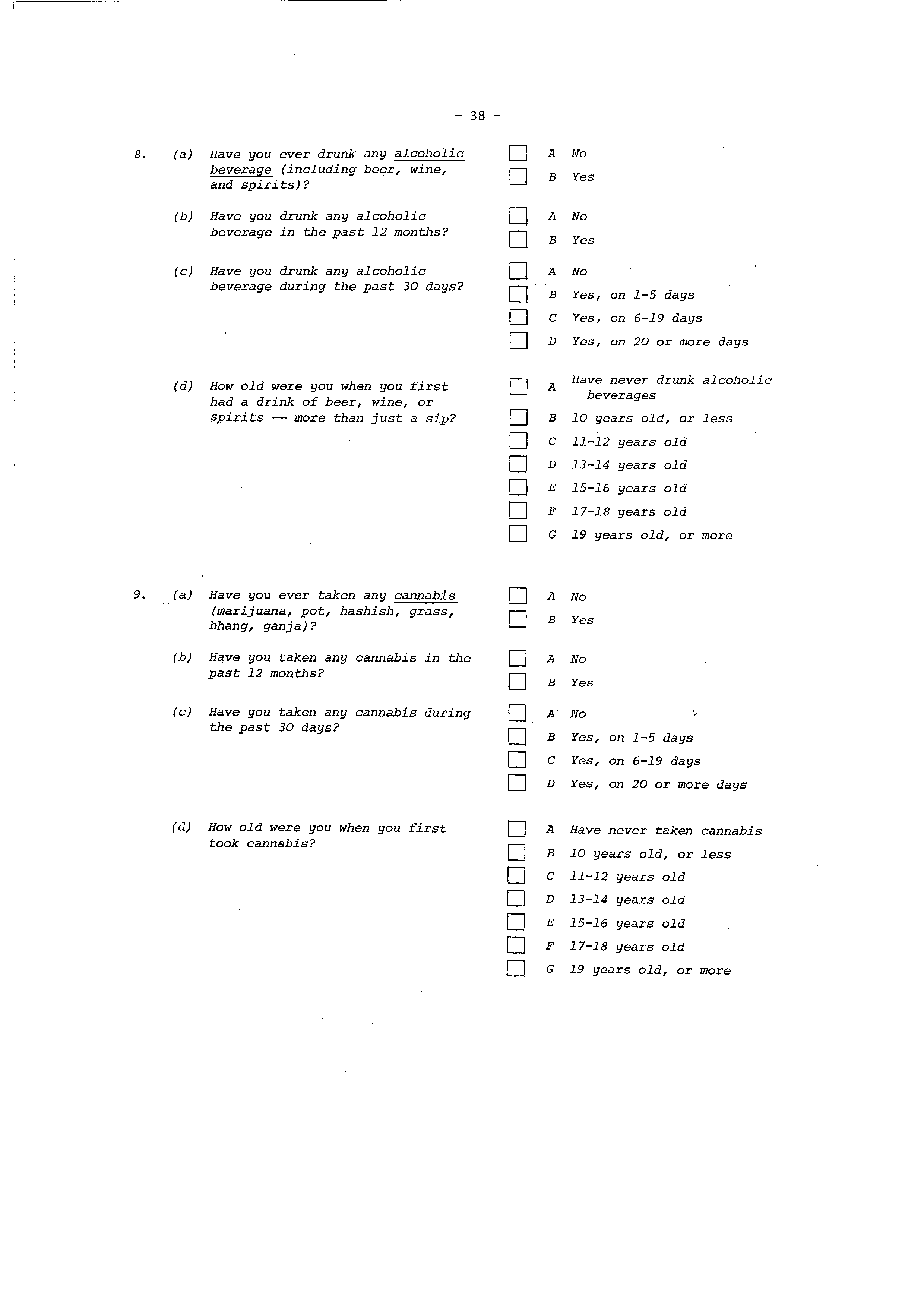

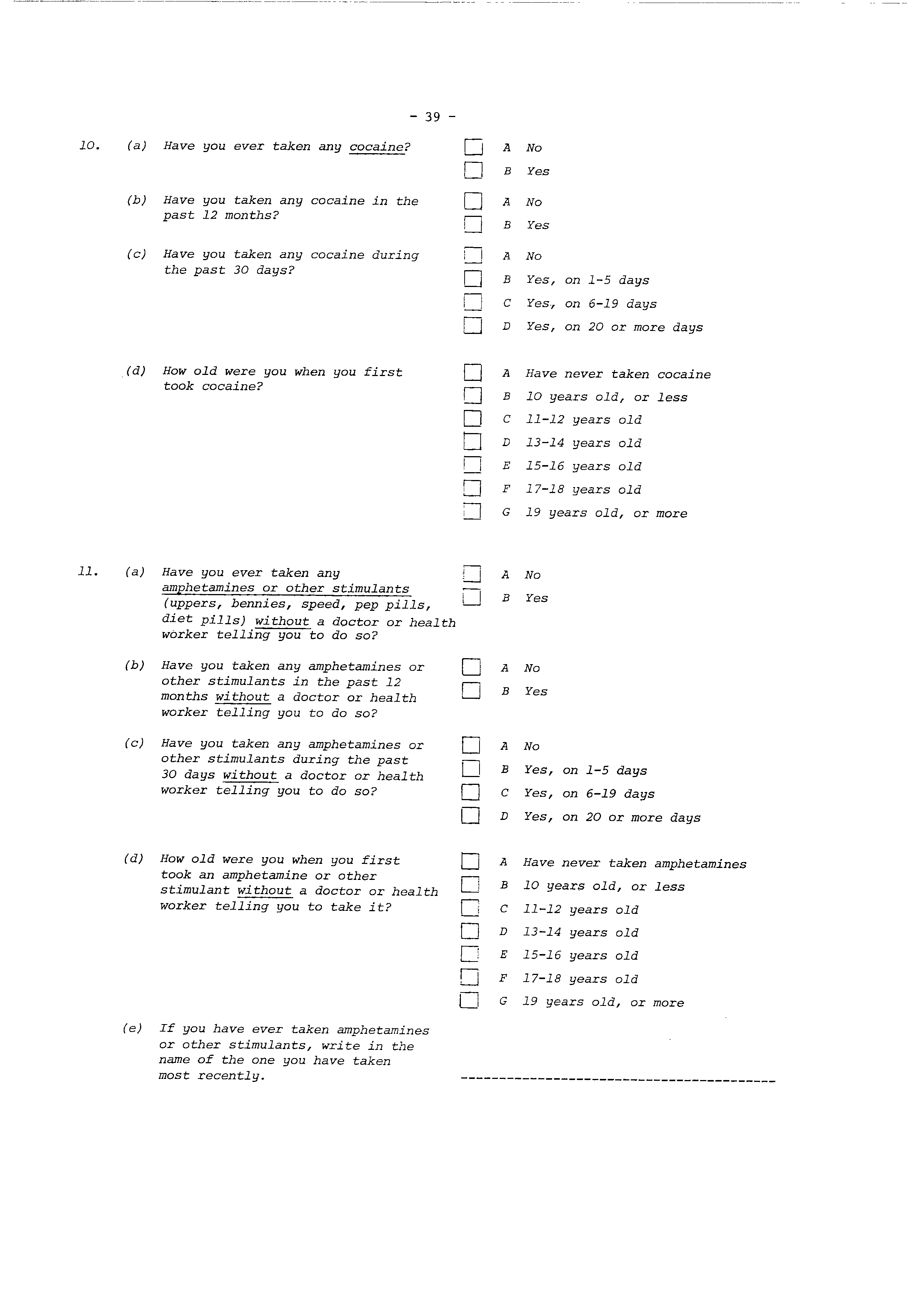

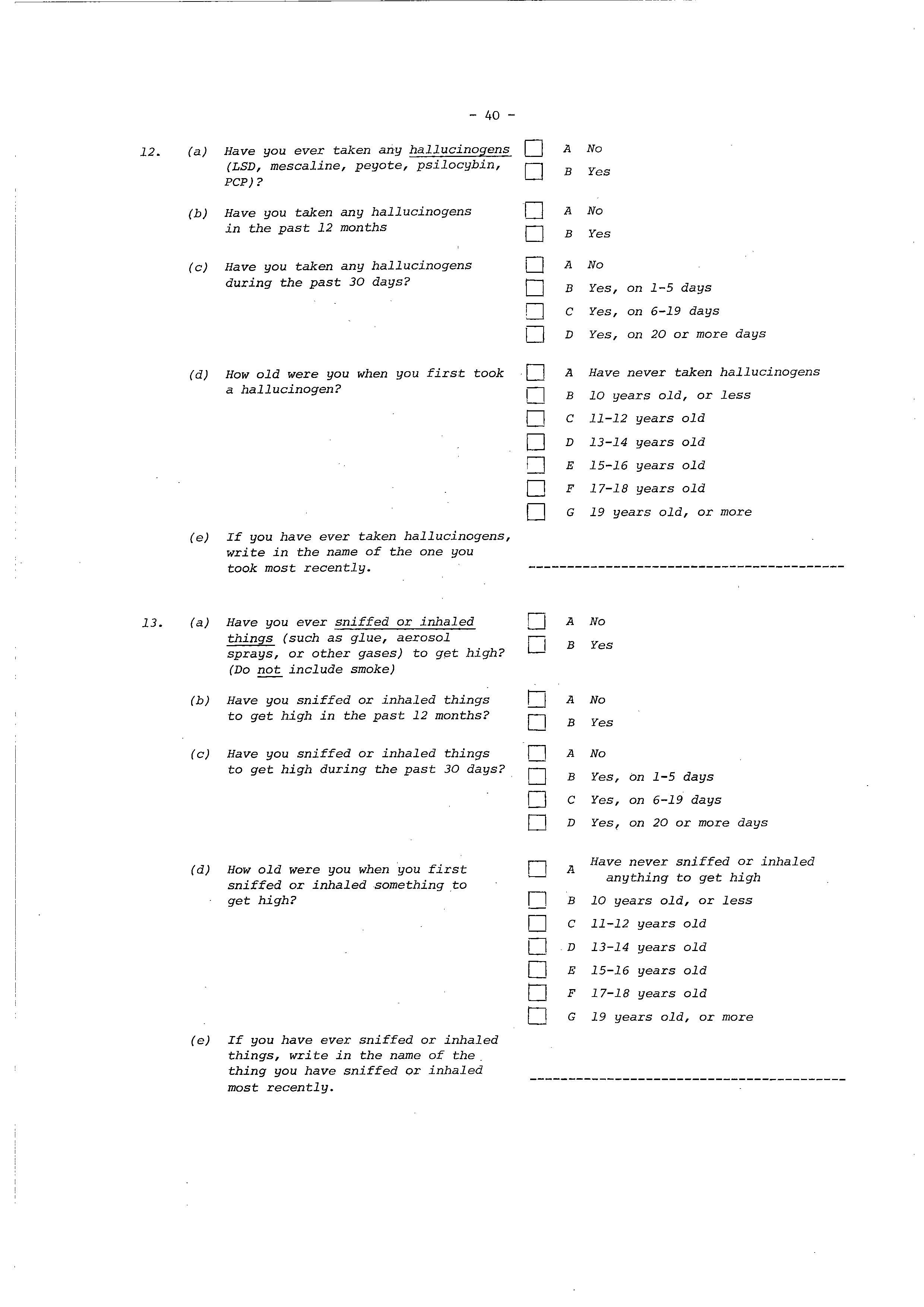

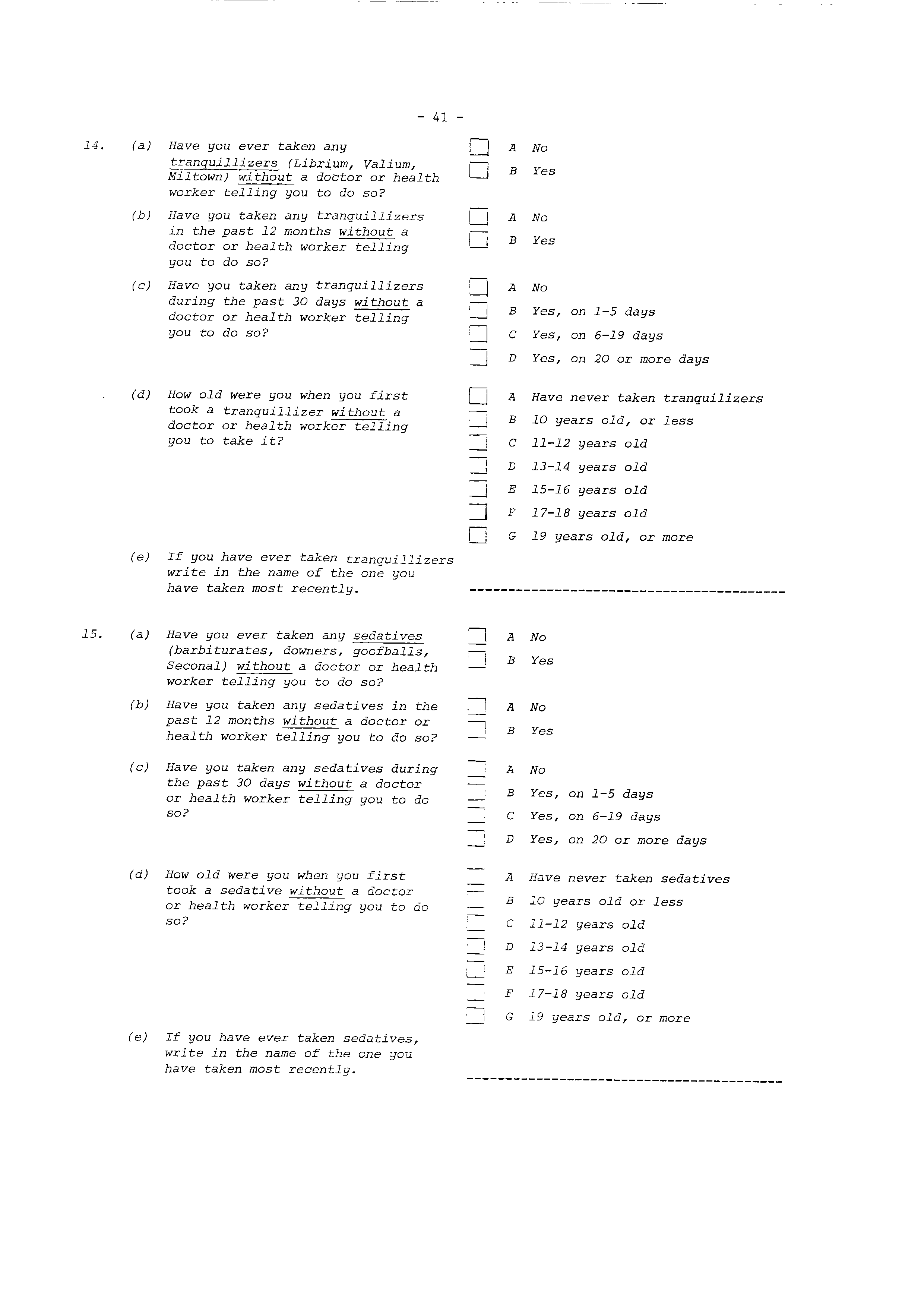

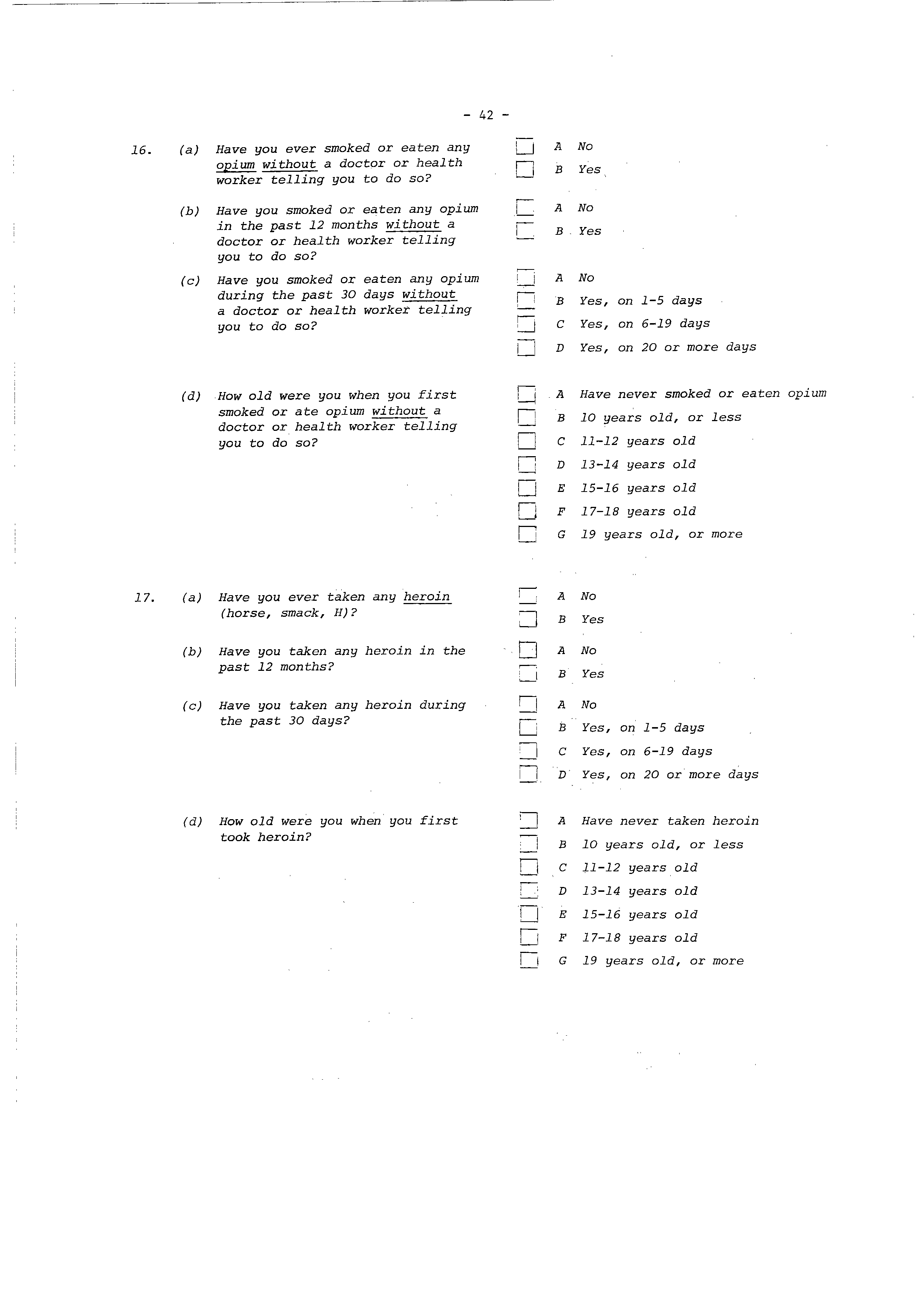

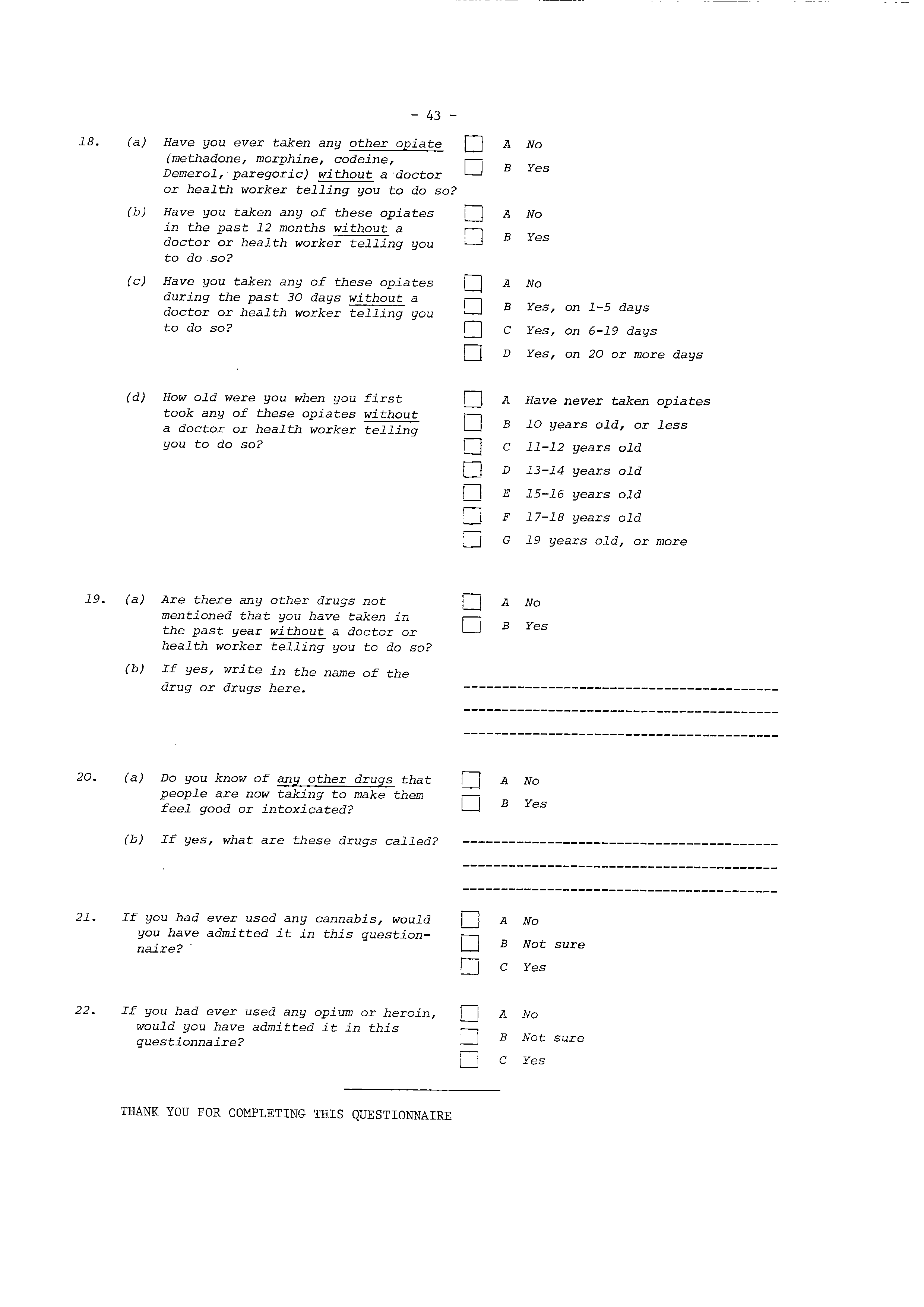

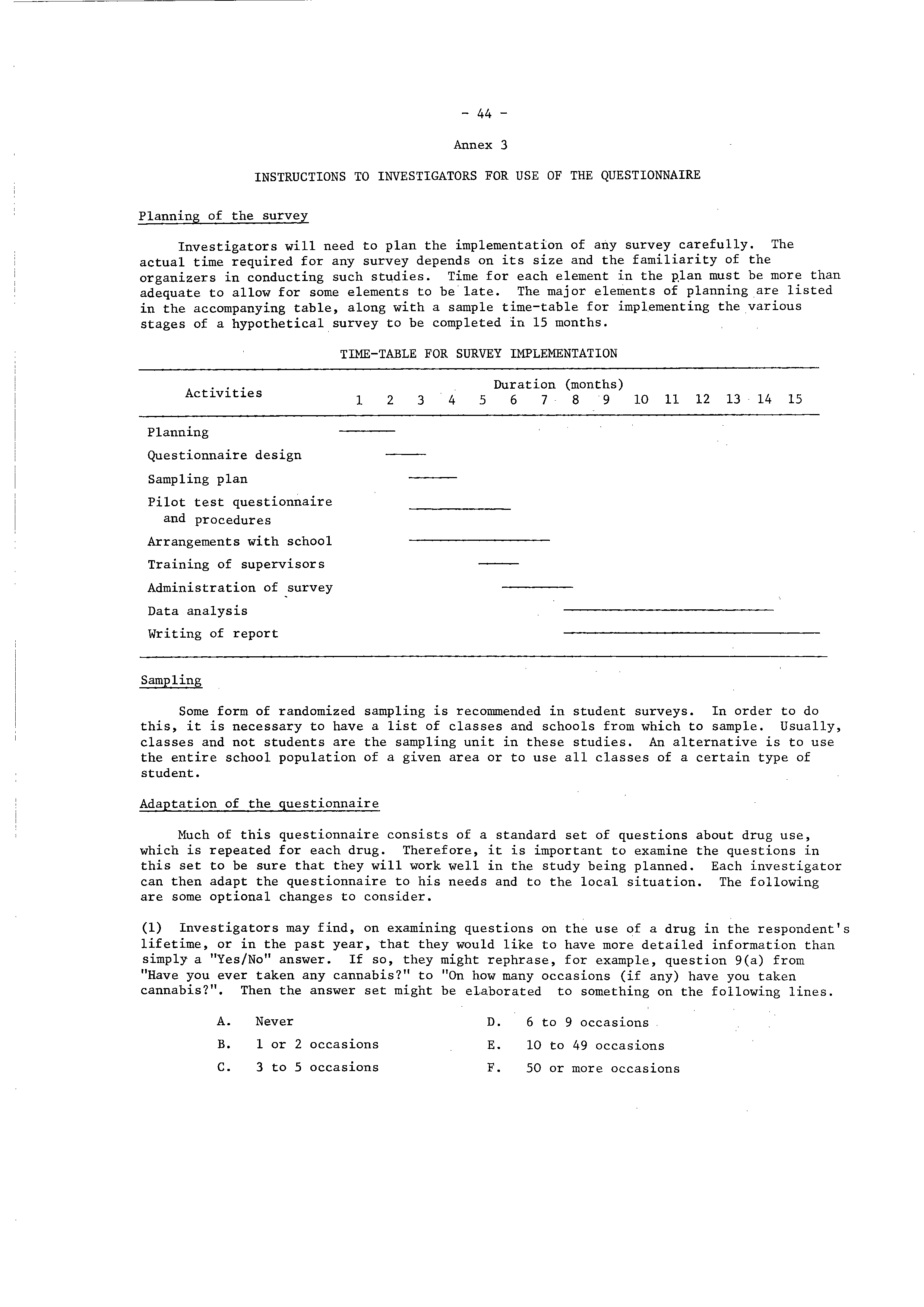

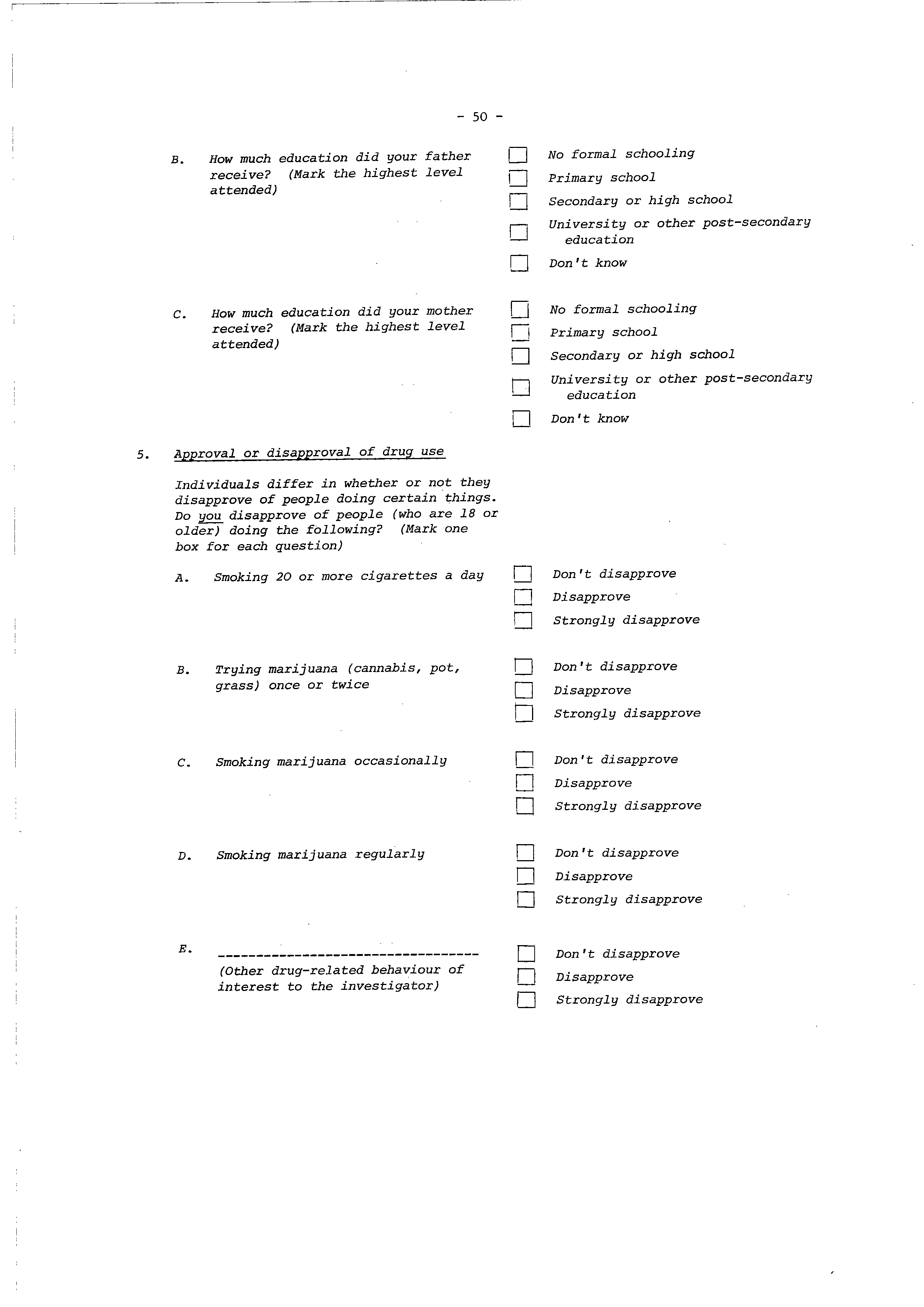

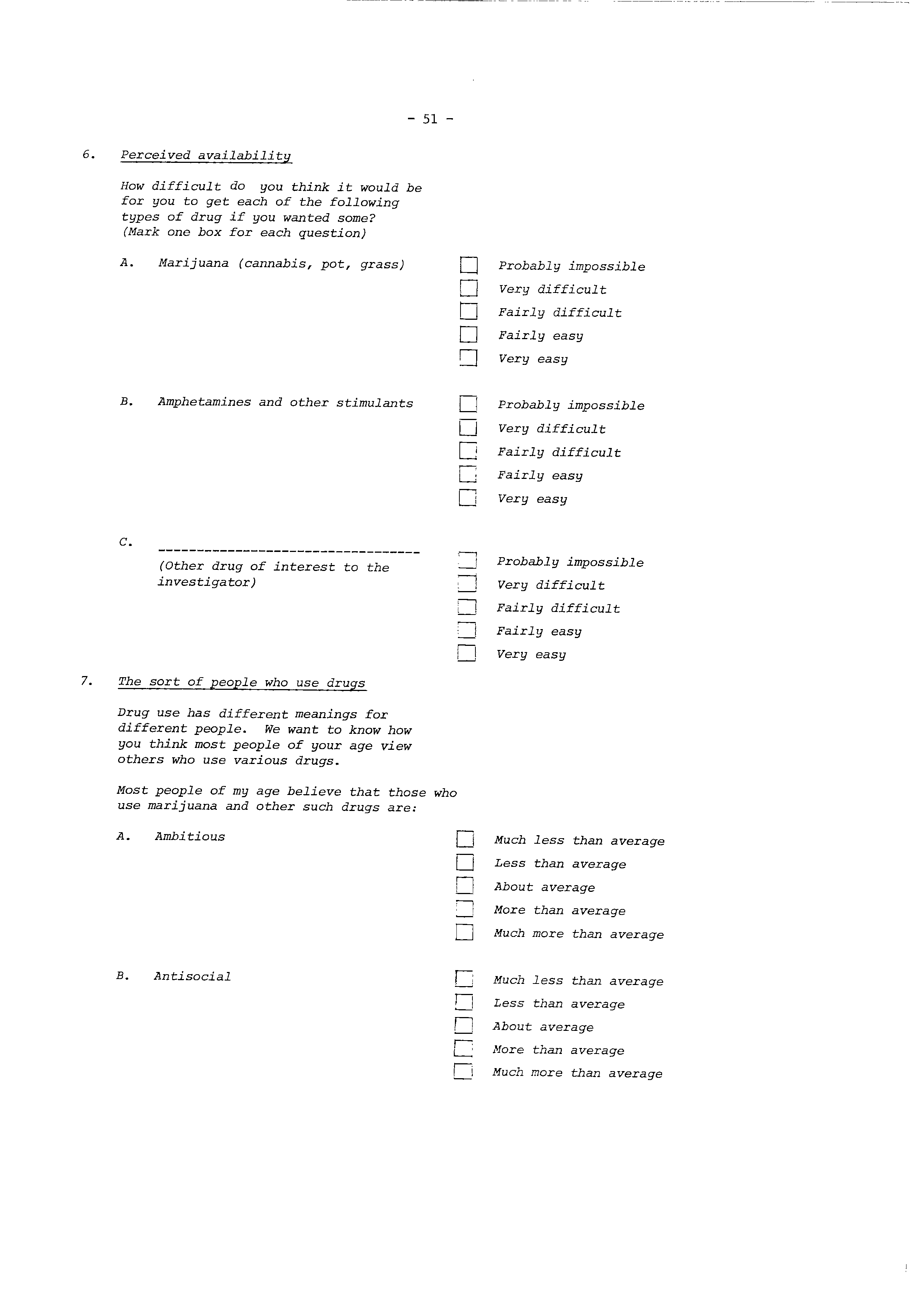

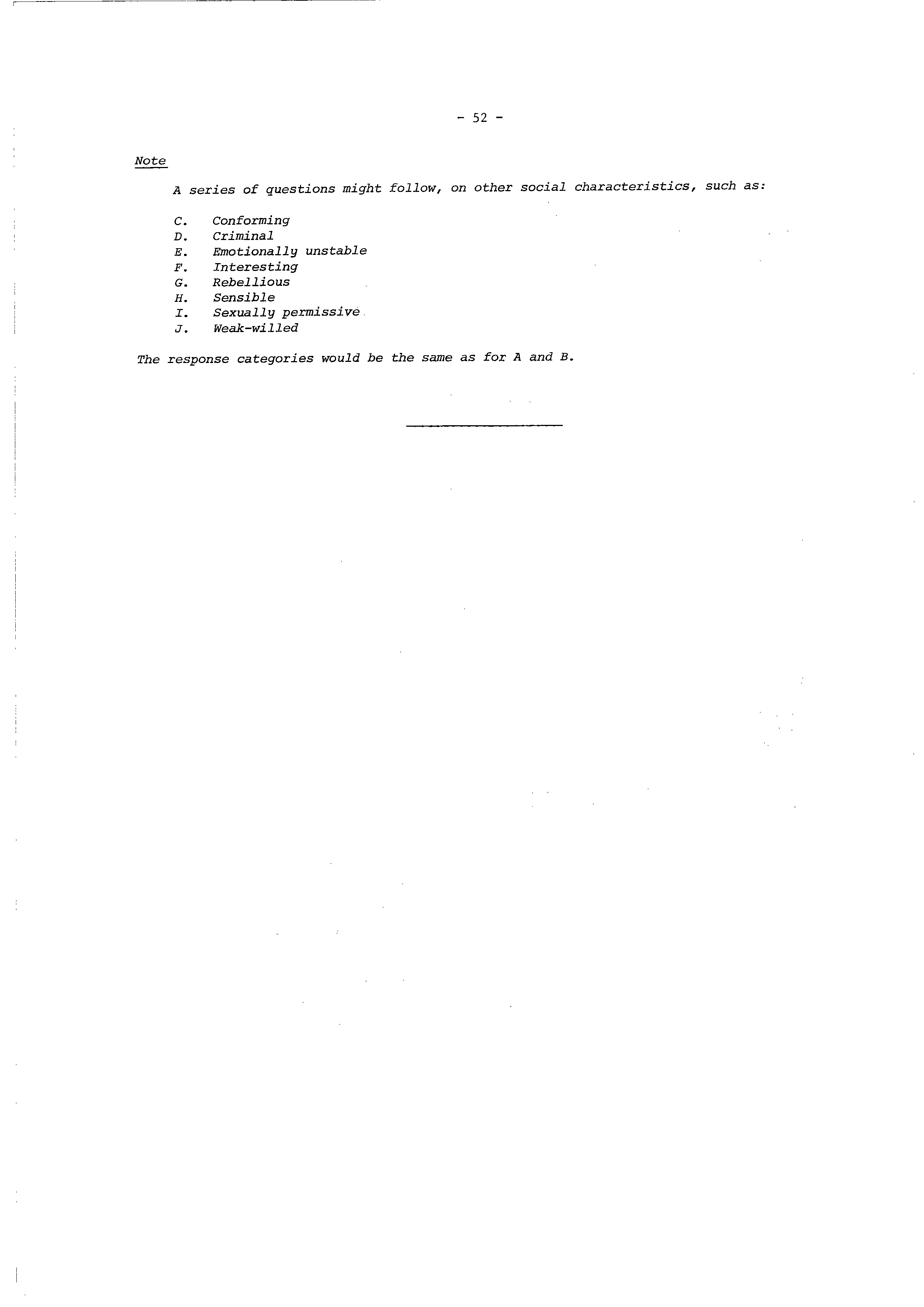

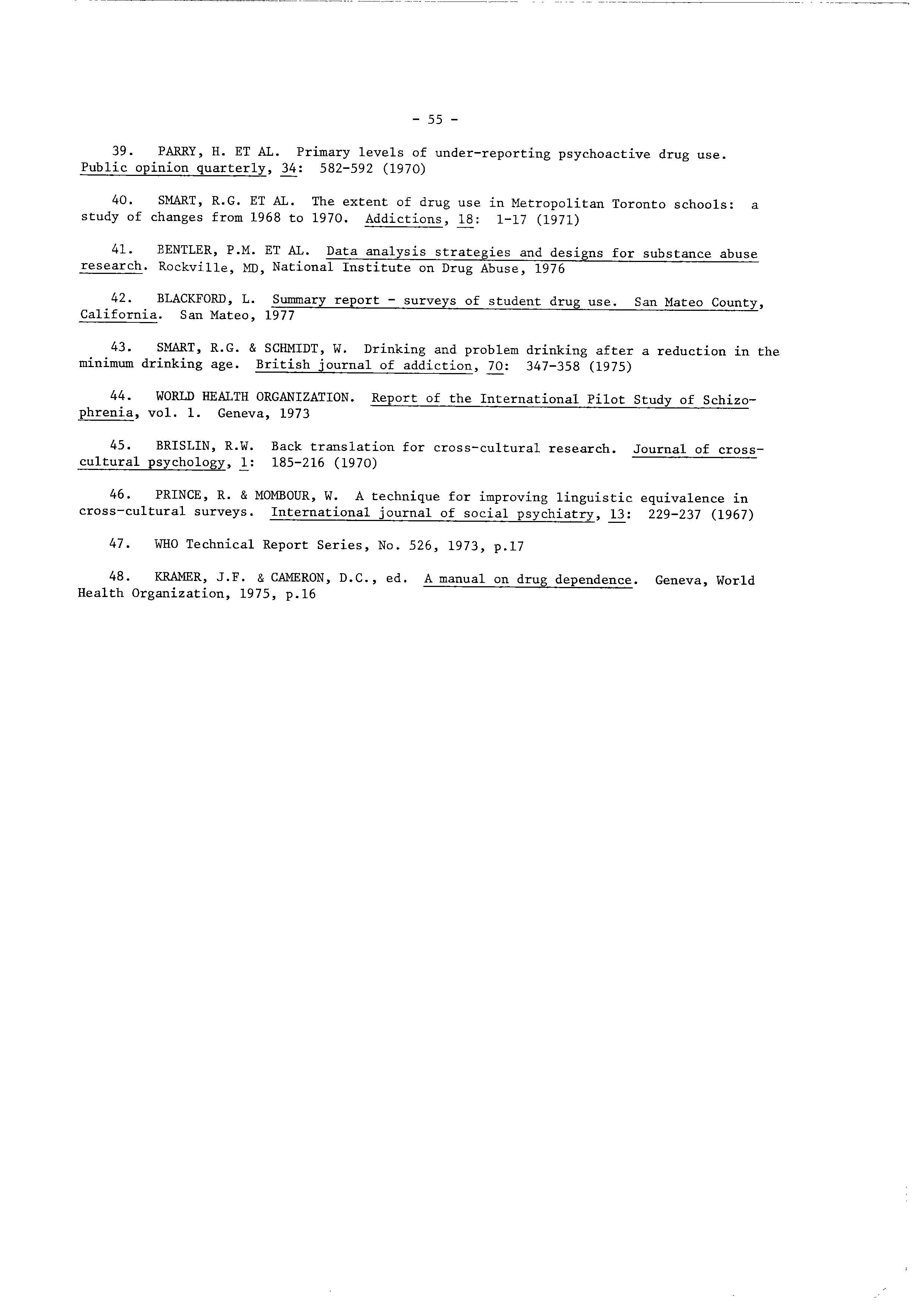

Supplement: Additional file 1: — WHO questionnaire, Geneva for substance use (DOCX 714 kb) [file 12887_2016_615_MOESM1_ESM.docx]
